# Vacuum sealing drainage system combined with an antibacterial jackfruit aerogel wound dressing and 3D printed fixation device for infections of skin soft tissue injuries

**DOI:** 10.1007/s10856-022-06709-9

**Published:** 2022-12-31

**Authors:** Xin Hu, Huijian Li, Wenting Guo, Huiqin Xiang, Liang Hao, Fanrong Ai, Souradeep Sahu, Chen Li

**Affiliations:** 1 Department of Orthopedic Surgery, The Second Affiliated Hospital of Nanchang University, Nanchang, Jiangxi 330006 People’s Republic of China; 2 The Second Clinical Medical School, Nanchang University, Nanchang, Jiangxi 330006 People’s Republic of China; 3 School of Mechanical & Electronic Engineering, Nanchang University, Nanchang, Jiangxi 330031 People’s Republic of China

## Abstract

Injuries and infections of skin and soft tissue are commonly encountered in primary health care and are challenging to manage. Vacuum sealing drainage (VSD) is generally used in clinical treatment, but current commercial methods of VSD have some disadvantages, such as easy blockage, nonantibacterial effects, and inconvenient curved surfaces. Herein, we report a functional zinc oxide/jackfruit aerogel (ZnO/JFA) composite material that is ultralight, superabsorbent and antibacterial as a new antibacterial VSD wound dressing. The JFA is carbonized from fresh jackfruit, and the JFA exhibits superhydrophilicity and superabsorbability. The water absorption rate of JFA was up to 1209.39%, and the SBF absorption rate was up to 1384.22%. The water absorption rate of ZnO/JFA was up to 494.47%, and the SBF absorption rate was up to 473.71%. The JFA and ZnO/JFA possess a pipeline structure, which is beneficial for absorbing wound exudates. In addition, surface modification of nanosized ZnO and its effects on antibacterial properties and biocompatibility were performed. When the concentration of ZnO/JFA was 3.125 mg/mL, the survival rate of human fibroblast cells was close to 80%, while the antibacterial rates against *Staphylococcus aureus, Pseudomonas aeruginosa* and *Escherichia coli* were up to 99.06%, 75.28% and 93.58%, respectively. Moreover, a 3D printed assisted device was introduced to make the ZnO/JFA wound dressing more attached to the bottom of the wound on a curved surface. An integrated device was formed under the printing mold, and then animal experiments were conducted in vivo. The results showed that a healing rate of almost 100% for infected skin wounds was obtained with this novel VSD device after 14 days, compared to only 79.65% without the VSD device. This novel VSD with a negative pressure suction dressing is beneficial for healing infectious wounds.

**Graphical Abstract:**

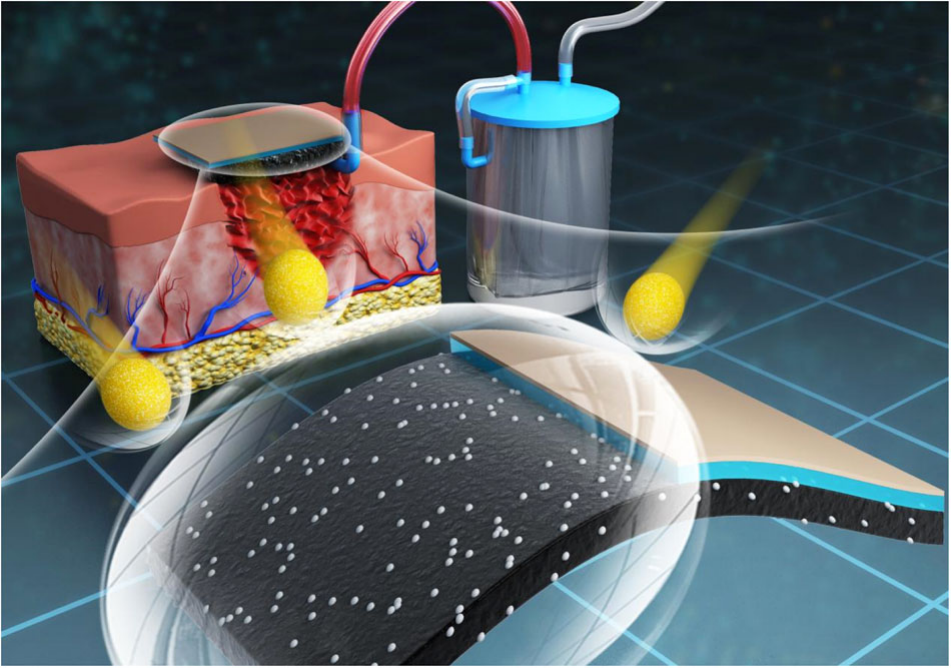

## Introduction

At present, injuries and infections of skin and soft tissue are still frequently encountered and challenging problems in the clinic [1]. Conventional treatment techniques easily cause the accumulation of exudates in wounds and thus create a more suitable microenvironment for bacterial growth, resulting in soft tissue empyema, edema, and poor blood circulation. The basic working principle of the VSD device is that a spongy suction material with negative pressure is used as the medium between the drainage tube and skin and the soft tissue injury or infected wound, and whole wound drainage can be achieved by connecting the drainage tube with the negative pressure device to remove the wound exudate and infected tissues in time. Continuous negative pressure can improve wound blood circulation, reduce tissue edema, and produce mechanical traction to promote the growth of granulation tissue [2, 3]. Closed dressings isolate the wound from the surrounding environment and effectively prevent the wound from being contaminated and infected [4]. At present, VSD has also achieved relatively good results in various clinically refractory skin diseases, soft tissue injuries, and skin grafting [5, 6]. However, infectious necrotic tissue may be adsorbed on spongy suction materials with a negative pressure, resulting in blockage [6]. Moreover, these spongy materials exhibit no antibacterial properties and may cause secondary infection. In addition, the traditional commercial VSD system does not conform to anatomical and physiological designs and is not suitable for curved surfaces [7].

In recent years, to prevent these shortcomings of VSD, researchers have tried to improve VSD in different aspects [8], especially in terms of spongy suction material with negative pressure. Aerogels are three-dimensional [9], ultralight [10], ultralow density [11, 12] carbon materials that can be used as adsorbents [13–16], catalysts, drug carriers [17–19], etc. In recent years, aerogels have attracted extensive attention in the field of medicine due to several characteristics, including high porosity [20], high specific surface area, wide sources, low cost and lack of toxicity [21], and their main application involves skin hemostasis and wound healing [22–25]. For example, Toribio et al. added grape skins and seed extracts to graphene oxide and chitosan aerogels. These aerogels can be used as hemostatic agents for wound management [26]. Zhang et al. embedded amino-MoS_2_ nanosheets into a chitosan aerogel and found that the aerogel could be used as an absorption and phototherapy agent to eliminate bacteria [27]. Because of their good performance, aerogels could be an ideal candidate for VSD adsorption devices to replace sponge materials for traditional negative pressure wound therapy devices. However, aerogels do not exhibit antibacterial effects [28] themselves. As a safe antibacterial material that was approved by the FDA, nanozinc oxide has been gradually used in clinical fields, such as stomatology and orthopedics [29, 30]. Therefore, modifying the nanozinc oxide surface of aerogel dressings is essential for inducing antibacterial properties.

In this study, a new ZnO/JFA with VSD compliant system device was developed. An ultralight, superabsorbent and antibacterial functional ZnO/JFA was prepared for a new antibacterial VSD wound dressing. The new VSD wound dressing was made suitable for curved and articular surfaces by utilizing a 3D printed assistant device according to the reconstruction of the wound model. Polylactic acid (PLA), an FDA-approved nontoxic material, was used to 3D print the assistant device in line with human anatomy. Then, the assistant device was wrapped on the upper surface of the designed aerogel dressing, making the new VSD system more efficient for curved surface wounds.

## Materials and methods

### Preparation of the nanosized ZnO surface-modified JFA

The JFA was prepared by a simple one-pot hydrothermal reaction. The JF was first cut into the appropriate volume and then put into a Teflon-lined stainless steel autoclave. After that, the autoclave was put into an oven and heated at 160 °C/8 h, 160 °C/10 h, 160 °C/12 h, 170 °C/10 h, and 180 °C/10 h. A black carbonaceous aerogel monolith was obtained after the hydrothermal reactions. The product was immersed in water and ethanol for several days to remove the soluble impurities. The corresponding carbonaceous aerogel was obtained after freeze drying. To prepare the ZnO/JFA, a piece of carbonaceous aerogel was fully immersed in a 200 mL aqueous solution containing 14.87 g of Zn(NO_3_)_2_·6(H_2_O) for 10 min. The mixture was heated to 65 °C, and 3.5 g of C_6_H_12_N_4_ was introduced into the liquid with stirring. After that, 5.71 mL of ammonia solution was added to the mixture, and the mixture was maintained at 65 °C for 10 min. Then, 0.12 g of trisodium citrate was added to the solution and incubated overnight in an oven at 85 °C. Finally, the above ZnO/carbonaceous aerogel was freeze dried, resulting in the nano-ZnO surface-modified jackfruit aerogel (ZnO/JFA).

### Characterization

The morphology of JFA and ZnO/JFA was observed by scanning electron microscopy (SEM, Zeiss, Germany). The water absorption ratio (%) of the aerogels was measured according to a previous report [31]. Before the measurement, the aerogels were dried in a vacuum oven at 50 °C. A certain weight of dry aerogel was immersed in deionized water and simulated body fluid (SBF, pH = 7.4) at 37 °C. After 24 h, the aerogels were removed, surface moisture was removed by filter paper, and then the aerogels were weighed. This process was repeated three times for each aerogel group. The water absorption ratio (%) was calculated with the following equation:

$${{{\mathrm{Water}}}}\,{{{\mathrm{absorption}}}}\,{{{\mathrm{ratio}}}}\,{{{\mathrm{(\% )}}}} = ({{{W}}}_{{{{\mathrm{swell}}}}} - {{{W}}}_{{{{\mathrm{dry}}}}})/{{{W}}}_{{{{\mathrm{dry}}}}} \times 100\%$$

where *W*_swell_ and *W*_dry_ are the weights of swollen aerogels and dry aerogels, respectively.

The tensile and stress−strain curves of the JFA aerogels were tested via a universal tester (CMT6104, MTS Systems Ltd, China) at a loading and unloading rate of 2 mm/min.

### In vitro biocompatibility

The cytotoxicity of ZnO/JFA was analyzed using a cell counting kit 8 (CCK-8). Sterile JFA and ZnO/JFA were immersed in the cell culture medium at a ratio of 200 mg material to 1 mL cell culture medium. Then, the mixture was cultured in a cell incubator containing 5% CO_2_ at 37 °C for 24 h, and extracts were collected and diluted in cell culture medium at ratios of 1/16, 1/32, 1/64, and 1/128. Human skin fibroblast (HSF) cells were inoculated into 96-well plates at a density of approximately 3500 cells per well and cultured with 100 μL of extracts at different concentrations. The extracts were changed every two days, and cell culture medium without extracts was used as a blank group. The HSF cells were cultured for 7 days, and the cell OD value of each well was measured by Synergy 2 (Biotek) at 450 nm according to the manufacturer’s instructions by the CCK-8 method.

### Antibacterial properties assay in vitro

The antibacterial properties of JFA and ZnO/JFA were analyzed. Normal Luria−Bertani (LB) medium was used as the control group. All samples were sterilized by ethylene oxide before testing. This study was processed against three of the most common clinical strains, *Staphylococcus aureus* (*S. aureus*, gram-positive, ATCC 25923), *Pseudomonas aeruginosa* (*P. aeruginosa*, gram-negative, ATCC 15442) and *Escherichia coli* (*E. coli*, gram-negative, CMCC44817). Three milliliters of LB broth and 100 μL of bacterial suspension were mixed in a sterile tube. Then, JFA and ZnO/JFA with different masses were added, and the specific groups were as follows: normal LB medium, 20 mg/mL, 30 mg/mL, and 40 mg/mL JFA immersion solution. Due to the addition of ZnO, we reduced the concentration of the material in the immersion solution, namely, the normal medium, and the concentrations of 2 mg/mL, 3 mg/mL, and 4 mg/mL ZnO/JFA immersion solution and cocultured in an orbital shaker for 6 h. After that, 50 μL of the coculture solution was removed and diluted 10^5^ times. Then, 50 μL of the media was used to coat a Petri dish, which was placed in a constant temperature incubator (37 °C) for 24 h. The plate counting method was used to compare the antibacterial potency of different samples.

### 3D printing method

First, a 60*30*3 printing model was established by CAD software, and then PLA was printed on an FDM printer.

### In vivo animal experiments

SD rats were purchased from Hunan Tianqin Company, and all animal procedures were performed according to the protocol approved by the Institutional Animal Care and Use Committee of Nanchang University (SYXK2020-0037). Suppurative infected wounds of rat models were used for in vivo wound healing experiments. Healthy female SD rats aged 8−12 weeks with similar body weights were used and divided into three groups, namely, the blank group, ZnO/JFA group and ZnO/JFA with VSD group. The round full-thickness cutaneous wound infected with a diameter of 10 mm was cut on one side of the back, and 100 μL of 10^8^/mL *S. aureus* solution was inoculated into the fascia layer to create a suppurative infection wound model. After inoculation for 24 h, suppurative infected wounds were caused. The wounds of rats in the blank group (povidone-iodine) were treated with a conventional dressing change. The wounds of rats in the ZnO/JFA group and ZnO/JFA with VSD group were covered with ZnO/JFA material and ZnO/JFA material with the VSD device and treated for one hour every day until the wound exudate was completely absorbed, respectively. All rats were sacrificed after 14 days, and a circle of skin around the wound was cut, fixed with paraformaldehyde and embedded in paraffin for HE staining and Masson staining.

## Results

### Physical properties of ZnO/JFA

**Fig. 1:**
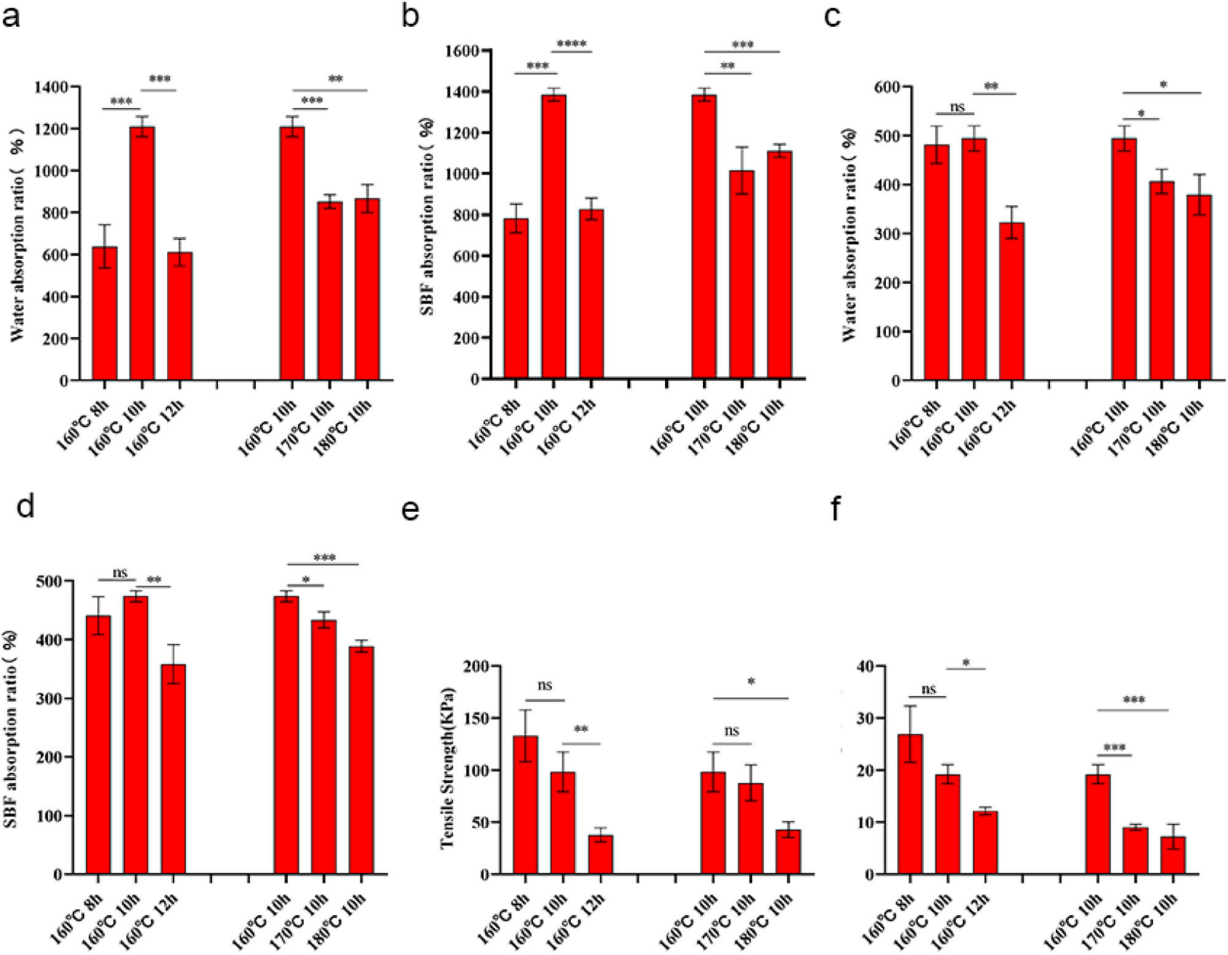
Absorption capacity and tensile capacity of JFA and ZnO/JFA. **a** Water absorption capacity of JFA, **b** SBF absorption capacity of JFA, **c** Water absorption capacity of ZnO/JFA, **d** SBF absorption capacity of ZnO/JFA, **e** Tensile capacity of JFA, **f** Tensile capacity of ZnO/JFA

JFA and ZnO/JFA were prepared by hydrothermal carbonization of nucleated jackfruit at different temperatures and times. Photographic images of the jackfruit aerogel are shown in Supplementary Fig. 1. Then, the adsorption properties and mechanical properties of the obtained JFA and ZnO/JFA were characterized. Due to the importance of adsorption performance, we first carried out adsorption experiments with JFA, as shown in Fig. 1a. Water absorption experiments with JFA revealed the following values: 638.56% of 160/8, 1209.39% of 160/10, 610.95% of 160/12, 852.46% of 170/10, and 867.27% of 180/10. To further conform to the human environment, an SBF adsorption experiment was carried out, as shown in Fig. 1b. SBF absorption experiments of JFA revealed that JFA hydrothermal carbonization at 160/10 showed the best absorption rates of water and SBF. Next, we carried out adsorption experiments of ZnO/JFA, Fig. 1c, d shows that ZnO/JFA hydrothermal carbonization at 160/10 showed the best absorption rates of water and SBF. We compared the adsorption rate of JFA with the adsorption rate of ZnO/JFA. JFA has better absorption, considering that the addition of nano-ZnO leads to a decrease in the porosity of JFA/ZnO, thus reducing the adsorption rate of JFA/ZnO. However, the composition of ZnO/JFA contains nano-ZnO, which exhibits better antibacterial properties (Supplement Table 1). After that, we carried out tensile tests with JFA and ZnO/JFA. As shown in Fig. 1e, f, the tensile test with JFA revealed that the tensile of 160/8 is 132.95 Kpa, the tensile of 160/10 is 98.36 Kpa, the tensile of 160/12 is 37.94 Kpa, the tensile of 170/10 is 87.78 Kpa, and the tensile of 180/10 is 42.85 Kpa. The tensile test of ZnO/JFA revealed that the tensile of 160/8 is 26.90 Kpa, the tensile of 160/10 is 19.23Kpa, the tensile of 160/12 is 12.17 Kpa, the tensile of 170/10 is 9.03 Kpa, and the tensile of 180/10 is 7.23 Kpa. We compared the tensile strength of JFA with the tensile strength of ZnO/JFA. It can be seen that JFA has better tensile strength, but the 3D-printed device can provide support (Supplementary Table 2).

**Fig. 2:**
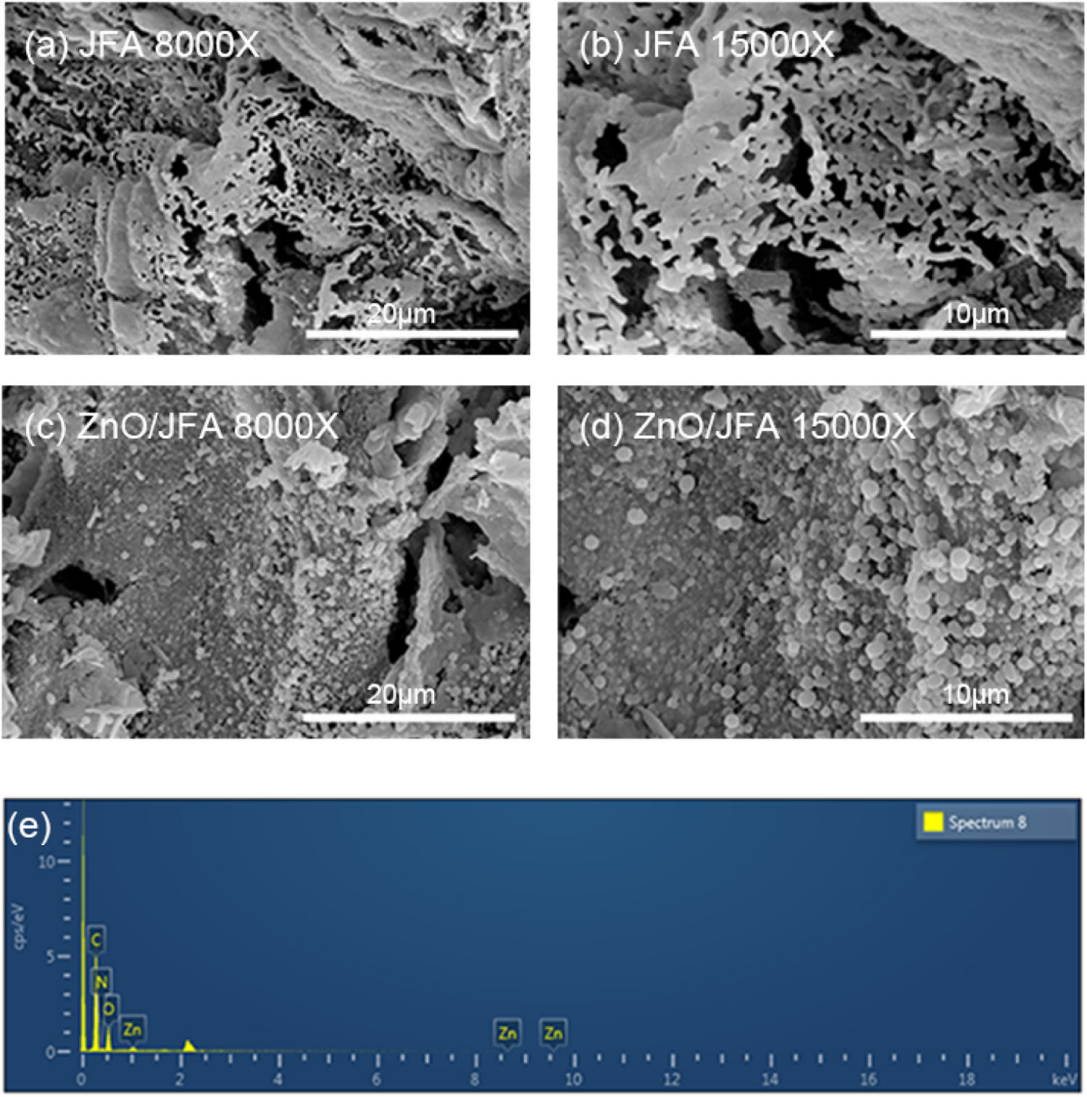
SEM images of JFA and ZnO/JFA at different magnifications: **a** MPC 8000X JFA, **b** MPC 15000X JFA, **c** MPC 8000X ZnO/JFA and **d** MPC 15000X ZnO/JFA. **e** Element analysis spectrum of the corresponding sample

In this new VSD wound dressing, the structure of jackfruit aerogel is important. The scanning electron microscopy (SEM) morphology of JFA and ZnO/JFA was investigated. Figure 2a–d shows that both JFA and ZnO/JFA present a porous mesh structure. Spherical nanosized ZnO particles were well dispersed on the surface of JFA. Energy dispersive spectrometry (EDS) of ZnO/JFA, as shown in Fig. 2e, further confirmed the presence of ZnO in JFA. It is worth noting that the porous mesh structure of the ZnO/JFA wound dressing is beneficial for body fluid absorption, while the presence of ZnO enables antibacterial properties.

### In vitro biocompatibility and antibacterial properties

**Fig. 3:**
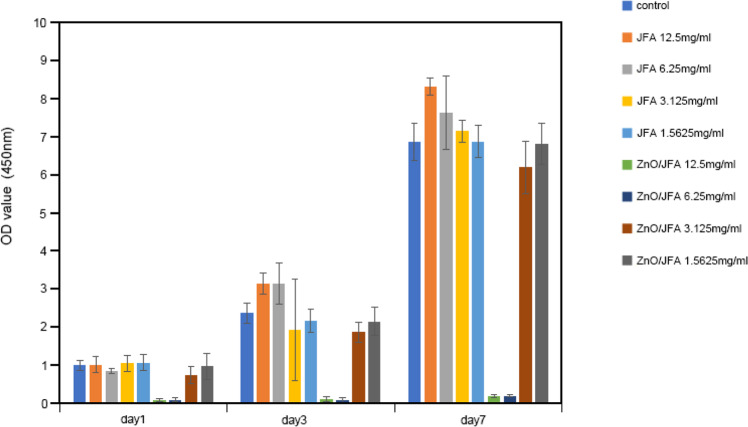
The OD values of the HSF concentration were measured at 1, 3, and 7 days in 12.5 mg/mL, 6.25 mg/mL, 3.125 mg/mL, and 1.5625 mg/mL JFA and 12.5 mg/mL, 6.25 mg/mL, 3.125 mg/mL, and 1.5625 mg/mL ZnO/JFA

Since JFA exhibits good biocompatibility, ZnO is the only nanosized antibacterial material certified by the U.S. Food and Drug Administration in vivo. The current cytotoxicity is mainly concentrated in ZnO/JFA biocompatibility. Herein, in vitro biocompatibility was evaluated with a cytotoxic test (CCK-8) for the JFA and ZnO/JFA samples. As shown in Fig. 3, the survival rate of human fibroblasts can be as high as 100% when the JFA concentration is 12.5 mg/mL. With a decrease in the JFA extract concentration, the survival rate of human fibroblasts was higher, indicating that JFA was less cytotoxic. The survival rate of human fibroblasts reached approximately 80% only when the concentration of ZnO/JFA was 3 mg/mL. With the increase in ZnO/JFA extract concentration, the survival rate of human fibroblasts was lower; when the concentration of ZnO/JFA was 12.5 mg/mL or 6.25 mg/mL, the survival rate of human fibroblasts was approximately 10%. Therefore, we can conclude that JFA exhibits low cytotoxicity and high biocompatibility. However, ZnO/JFA exhibits high cytotoxicity and low biocompatibility; thus, further investigation is necessary.

**Fig. 4:**
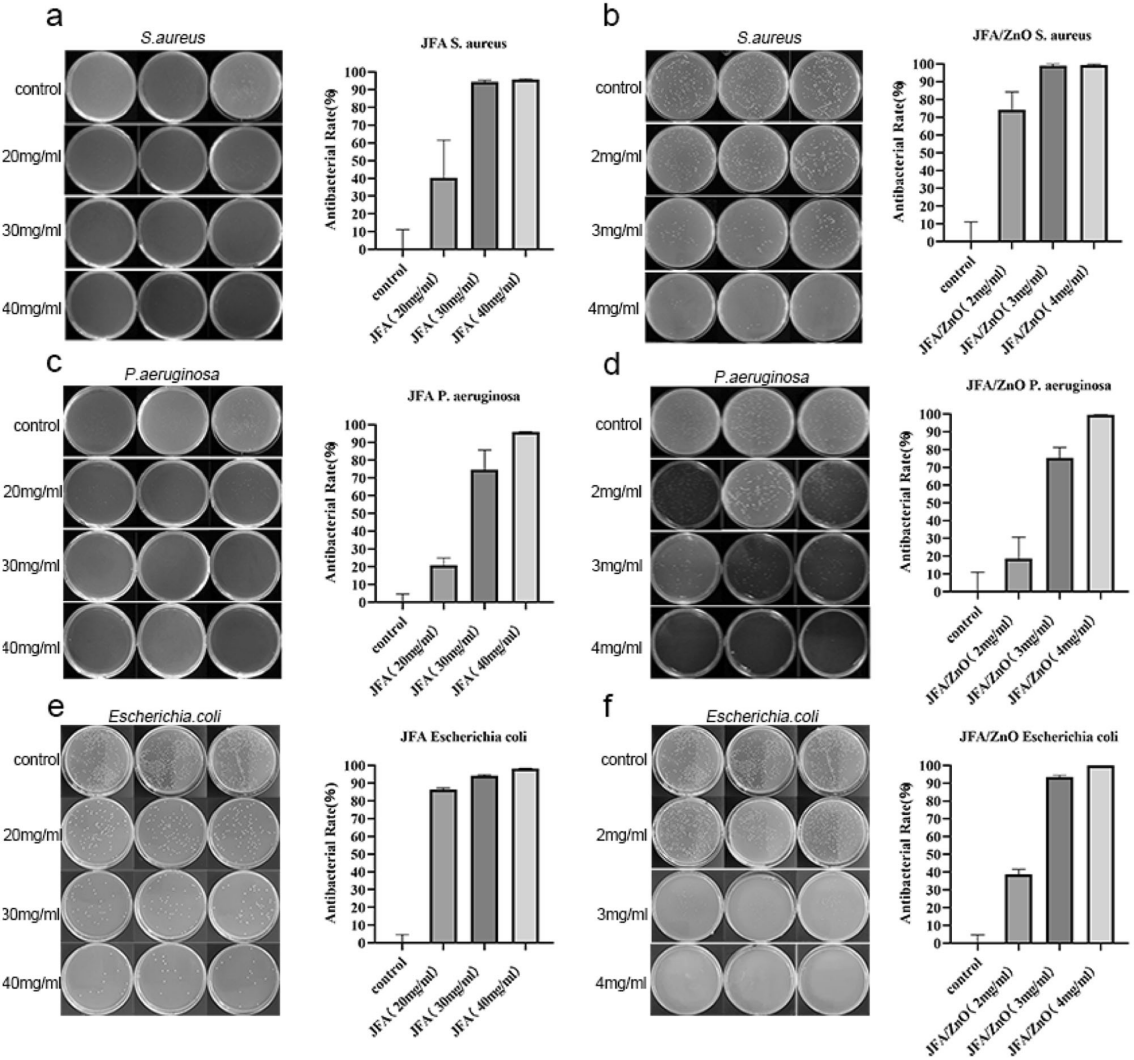
**a** Growth diagram for the antibacterial capacity of JFA soaking solution against *S. aureus* at concentrations of 20 mg/mL, 30 mg/mL and 40 mg/mL; **b** growth diagram for the antibacterial capacity of 2 mg/mL, 3 mg/mL and 4 mg/mL ZnO/JFA immersion solution against *S. aureus*; **c** growth diagram for the antibacterial capacity of 20 mg/mL, 30 mg/mL and 40 mg/mL JFA immersion solution against *P. aeruginosa*; **d** growth diagram for the antibacterial capacity of 2 mg/mL, 3 mg/mL, 4 mg/mL ZnO/JFA immersion solution against *P. aeruginosa*; **e** growth diagram for the antibacterial capacity of JFA soaking solution against *E. coli* at concentrations of 20 mg/mL, 30 mg/mL and 40 mg/mL; and **f** growth diagram for the antibacterial capacity of 2 mg/mL, 3 mg/mL and 4 mg/mL ZnO/JFA immersion solution against *E. coli*

To further explore the antibacterial properties of ZnO/JFA, we carried out antibacterial experiments. As shown in Fig. 4a, after the addition of JFA immersion solution for coculture, the JFA concentration of the extract was 0 mg/mL, 10 mg/mL, 20 mg/mL, and 30 mg/mL, the inhibitory rates of *S. aureus* were 0%, 40.25%, 94.34%, and 95.60%, respectively, and the number of *S. aureus* decreased more with increasing JFA concentration. JFA has an obvious inhibitory effect on *S. aureus* at a certain concentration. Next, we further discussed the antibacterial performance of ZnO/JFA. Due to the addition of ZnO, the mass fraction of ZnO/JFA was reduced, but the number of *S. aureus* was also reduced and stronger than that of JFA, as shown in Fig. 4b. The ZnO/JFA concentrations of the extract were 0 mg/mL, 1 mg/mL, 2 mg/mL, and 3 mg/mL, and the inhibitory rates of *S. aureus* were 0%, 74.21%, 99.06%, and 99.37%, respectively, indicating that ZnO/JFA had a more significant inhibitory effect on the growth of *S. aureus*. Similarly, we also adopted the same conditions to test the antibacterial effect of JFA and ZnO/JFA on the gram-negative bacteria *P. aeruginosa* and *E. coli*, as shown in Fig. 4c, e. JFA also has an inhibitory effect on the growth of *P. aeruginosa* and *E. coli* at the same mass fraction of materials. Then, we conducted the next experiment to verify whether ZnO/JFA has the same inhibitory effect on the growth of *P. aeruginosa* and *E. coli*. As shown in Fig. 4d, f, ZnO/JFA also has a significant inhibitory effect on the growth of *P. aeruginosa* and *E. coli*, which is stronger than that of JFA. In conclusion, our results indicate that both JFA and ZnO/JFA exhibit strong antibacterial activity against gram-positive bacteria and gram-negative bacteria, especially ZnO/JFA.

### Wound healing of animal skin surface infections

**Fig. 5:**
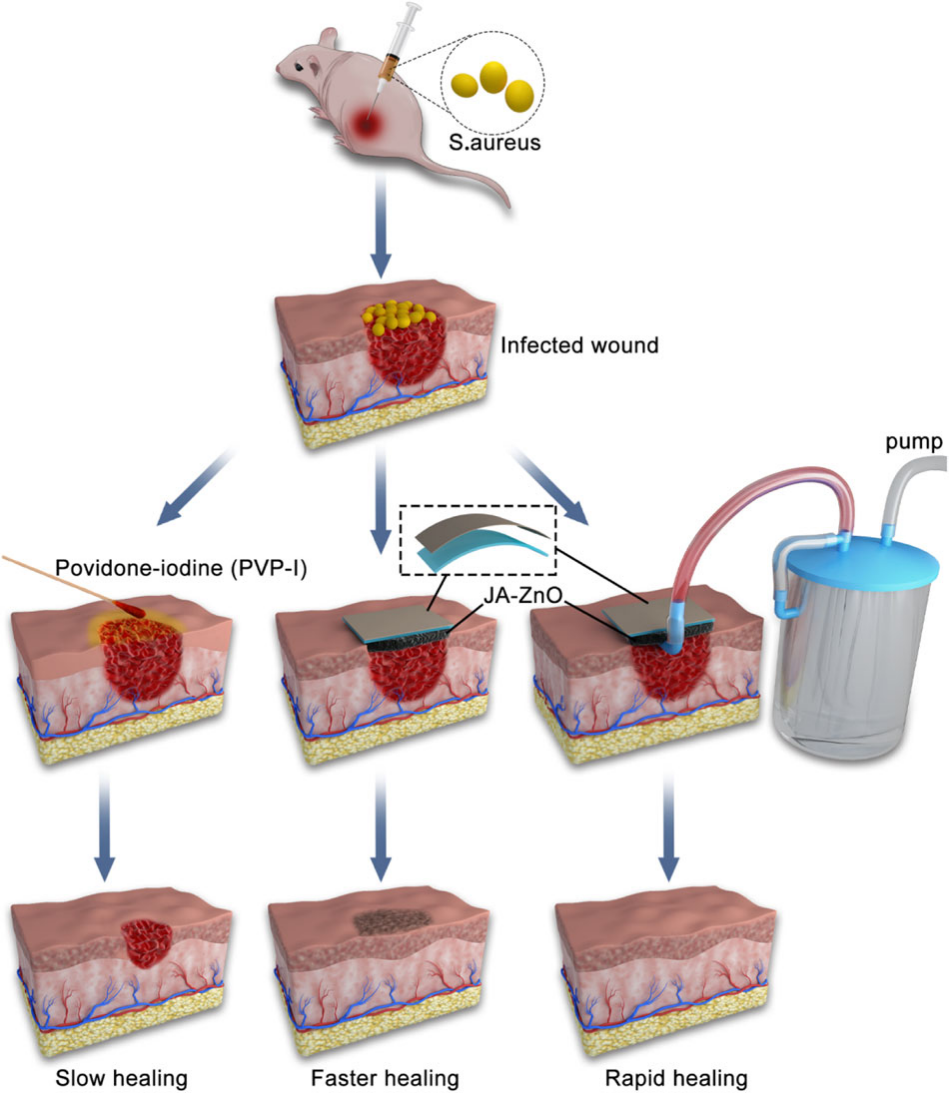
Mechanism diagram of povidone-iodine (PVP-I), ZnO/JFA, and ZnO/JFA with VSD treatment in evaluating the healing process of infected skin wounds in rats

Figure 5 shows the mechanism diagram of three different healing processes for infected skin wounds of rats, and these processes are povidone-iodine (PVP-I), ZnO/JFA, and ZnO/JFA with VSD treatment. For the infected wound, ZnO/JFA with VSD should effectively promote the healing of skin and soft tissue injury and infection.

**Fig. 6:**
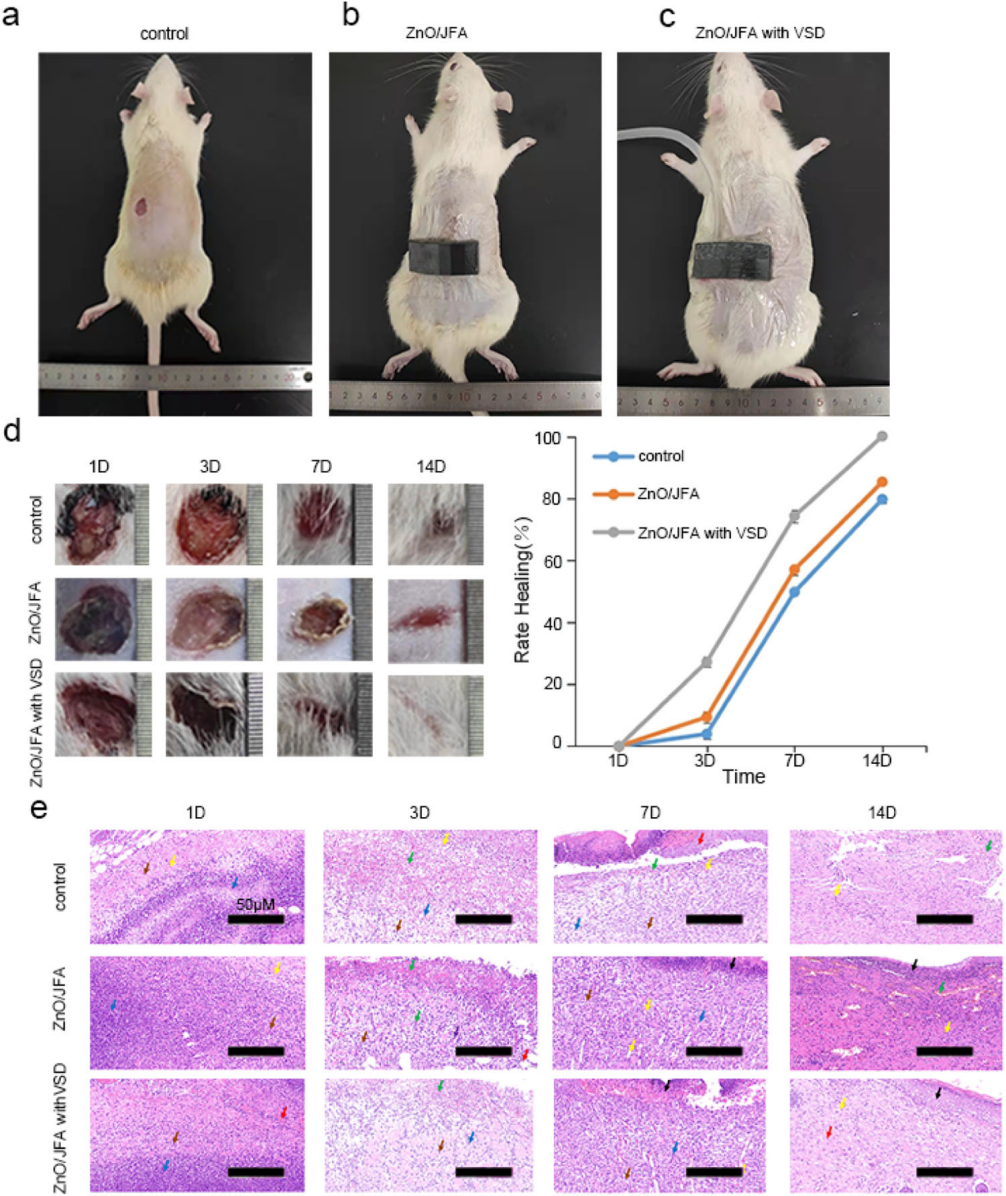
Therapeutic effect evaluation of JFA, ZnO/JFA, and ZnO/JFA with VSD using a mouse skin model. Mouse skin was treated with povidone-iodine (PVP-I) as a blank control and **a** blank control; **b** ZnO/JFA and **c** ZnO/JFA with VSD. **d** Representative image of the purulent infected wound and quantitative analysis of the healing rate. **e** Histopathological analysis after 1 D, 3 D, 7 D, and 14 D

Next, we further studied the healing ability of ZnO/JFA with VSD on infected skin wounds in vivo. First, we constructed a rat model of skin infection wounds and treated them in different ways. In Fig. 6a, only povidone-iodine (PVP-I) was adopted; in Fig. 6b, ZnO/JFA was applied; in Fig. 6c, ZnO/JFA with VSD was applied; and the wound healing results were observed on Days 1, 3, 7, and 14. As shown in Fig. 6d, the wound healing of the rats with dressing change alone was slow, and large residual wounds were observed. However, compared to the rats with ZnO/JFA, the rats with ZnO/JFA with VSD exhibited faster wound healing. Only faint scarring was observed at the original wound of the rats. Hematoxylin and eosin (H&E) staining, as shown in Fig. 6e, demonstrated that ZnO/JFA with VSD also promoted wound healing. In summary, our results indicate that ZnO/JFA with VSD can promote the healing of skin-infected wounds to a certain extent.

## Discussion

Excessive infection exudation is still the main reason for the lack of efficacy in conventional dressing change therapy. In addition, traditional VSD is easy to block, and the dressings exhibit no antibacterial properties, do not conform to human physiological anatomy and are not suitable for joints. To overcome these shortcomings, we were inspired by the strong adsorption ability of jackfruit aerogels and developed a new VSD system of jackfruit aerogels that are loaded with nanozinc oxide by 3D printing technology. Further experimental results show that the proposed ZnO/JFA not only improves adsorption properties but also provides antibacterial capability and excellent biocompatibility. Moreover, compared with traditional VSD devices, the new ZnO/JFA VSD devices are less expensive and easier to obtain. Therefore, replacing the dressings continually is more acceptable for patients. In addition, the device also has some problems that need to be further studied. For example, ZnO/JFA/160/10 does not exhibit the best tensile properties, and whether other materials can be embedded to achieve the best properties must be studied. Therefore, before further clinical application, it is necessary to conduct a systematic study of the ultrastrong adsorption multifunctional aerogel combined with 3D printing to produce an antibacterial ultralight vacuum sealing drainage device.

## Supplementary information

Supporting information
