# Supplementary material for: Vacuum sealing drainage system combined with an antibacterial jackfruit aerogel wound dressing and 3D printed fixation device for infections of skin soft tissue injuries
Source: J Mater Sci Mater Med. 2022 Dec 31;34(1):1. doi: 10.1007/s10856-022-06709-9 (PMC9805414; doi:10.1007/s10856-022-06709-9)
Supplement: Supplementary file 1 — Supporting information [file 10856_2022_6709_MOESM1_ESM.docx]

Supporting information

[Supplement](javascript:;) figures and tables

Figures


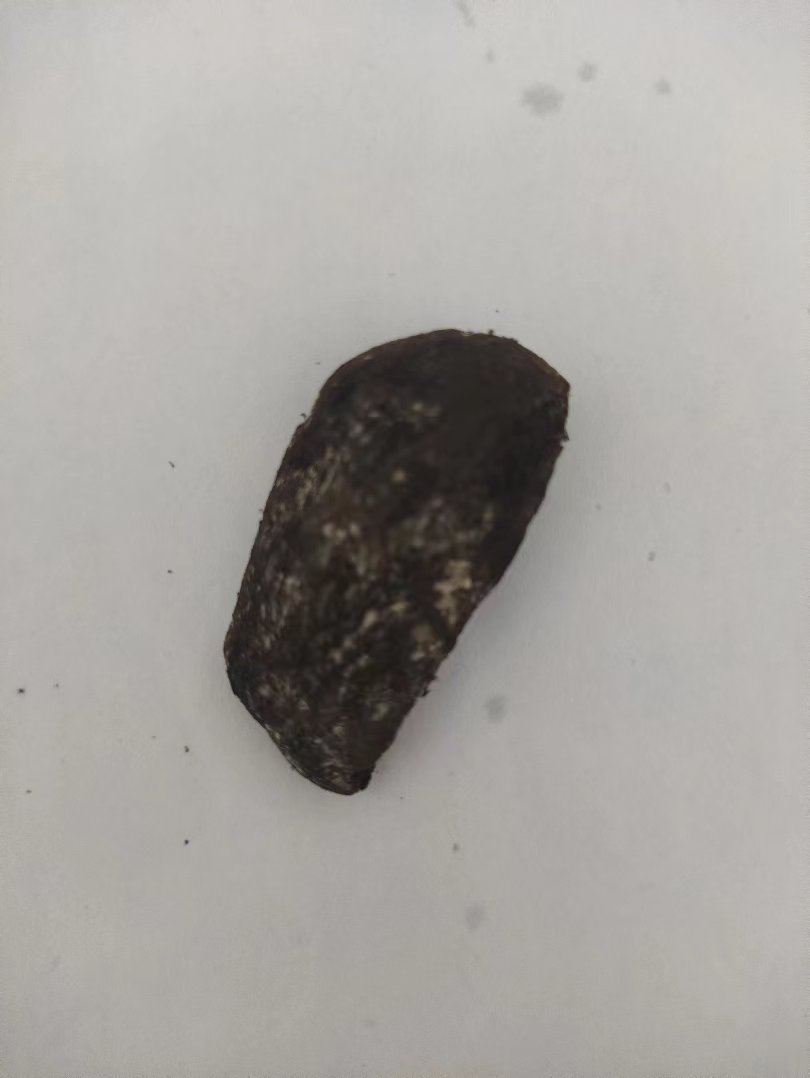


Figure 1. photographic images of the Jackfruit aerogel.

Tables

| **Water absorption experiment of JFA** | | | | | | | | | |
| --- | --- | --- | --- | --- | --- | --- | --- | --- | --- |
| **160℃ 8h** | | **160℃ 10h** | | **160℃ 12h** | | **170℃ 10h** | | **180℃ 10h** | |
| Wdry | Wswell | Wdry | Wswell | Wdry | Wswell | Wdry | Wswell | Wdry | Wswell |
| 0.0805 | 0.5036 | 0.0825 | 1.1232 | 0.0747 | 0.5047 | 0.0849 | 0.8382 | 0.0794 | 0.7508 |
| 0.0745 | 0.5676 | 0.0880 | 1.1132 | 0.0866 | 0.5802 | 0.0851 | 0.7845 | 0.0729 | 0.8518 |
| 0.0805 | 0.6667 | 0.0813 | 1.0583 | 0.0729 | 0.5739 | 0.0800 | 0.7586 | 0.0832 | 0.9704 |
| 638.56% | | 1209.39% | | 610.95% | | 852.46% | | 867.27% | |
| **SBF absorption experiment of JFA** | | | | | | | | | |
| **160℃ 8h** | | **160℃ 10h** | | **160℃ 12h** | | **170℃ 10h** | | **180℃ 10h** | |
| Wdry | Wswell | Wdry | Wswell | Wdry | Wswell | Wdry | Wswell | Wdry | Wswell |
| 0.0848 | 0.6831 | 0.0786 | 1.1383 | 0.0798 | 0.7105 | 0.0798 | 0.8349 | 0.0775 | 0.9655 |
| 0.0817 | 0.7342 | 0.0758 | 1.1343 | 0.0740 | 0.6696 | 0.0758 | 0.7965 | 0.0893 | 1.0583 |
| 0.0821 | 0.7731 | 0.0750 | 1.1310 | 0.0796 | 0.7867 | 0.0728 | 0.9073 | 0.0791 | 0.9498 |
| 781.95% | | 1384.22% | | 827.84% | | 1014.44% | | 1110.56% | |
| **Water absorption experiment of ZnO/JFA** | | | | | | | | | |
| **160℃ 8h** | | **160℃ 10h** | | **160℃ 12h** | | **170℃ 10h** | | **180℃ 10h** | |
| Wdry | Wswell | Wdry | Wswell | Wdry | Wswell | Wdry | Wswell | Wdry | Wswell |
| 0.082 | 0.441 | 0.0620 | 0.3540 | 0.059 | 0.241 | 0.0520 | 0.2500 | 0.0450 | 0.2200 |
| 0.083 | 0.494 | 0.0650 | 0.3840 | 0.06 | 0.24 | 0.0410 | 0.2170 | 0.0470 | 0.2040 |
| 0.067 | 0.409 | 0.0600 | 0.3730 | 0.065 | 0.299 | 0.0530 | 0.2700 | 0.0470 | 0.2420 |
| 481.14% | | 494.47% | | 322.82% | | 406.49% | | 379.28% | |
| **SBF absorption experiment of ZnO/JFA** | | | | | | | | | |
| **160℃ 8h** | | **160℃ 10h** | | **160℃ 12h** | | **170℃ 10h** | | **180℃ 10h** | |
| Wdry | Wswell | Wdry | Wswell | Wdry | Wswell | Wdry | Wswell | Wdry | Wswell |
| 0.062 | 0.339 | 0.0450 | 0.2630 | 0.101 | 0.424 | 0.0550 | 0.2860 | 0.0760 | 0.3630 |
| 0.081 | 0.41 | 0.0530 | 0.3010 | 0.072 | 0.346 | 0.0590 | 0.3230 | 0.0830 | 0.4080 |
| 0.085 | 0.484 | 0.0480 | 0.2730 | 0.065 | 0.308 | 0.0600 | 0.3200 | 0.0640 | 0.3180 |
| 440.79% | | 473.71% | | 358.07% | | 433.60% | | 388.69% | |

Table1：Absorption experiments

|  | **Tensile experiment of JFA** | | | | |
| --- | --- | --- | --- | --- | --- |
|  | **160℃ 8h** | **160℃ 10h** | **160℃ 12h** | **170℃ 10h** | **180℃ 10h** |
| Tensile(kPa)1 | 21.52306835 | 21.27677567 | 12.66728567 | 9.555964295 | 5.544871092 |
| Tensile(kPa)2 | 32.33025624 | 18.56180393 | 11.66693072 | 8.391183998 | 8.90825734 |
| Tensile(kPa)3 | 26.85286601 | 17.84581343 |  | 9.132733941 |  |
|  | **Tensile experiment of ZnO/JFA** | | | | |
|  | **160℃ 8h** | **160℃ 10h** | **160℃ 12h** | **170℃ 10h** | **180℃ 10h** |
| Tensile(kPa)1 | 133.3571934 | 97.85800454 | 38.11910725 | 87.7888214 | 43.14226591 |
| Tensile(kPa)2 | 107.7782804 | 117.6166369 | 44.50162085 | 105.0456448 | 50.27492071 |
| Tensile(kPa)3 | 157.7217723 | 79.62142474 | 31.19536565 | 70.5200651 | 35.12899681 |

Table2: Tensile experiments
